# Understanding the Impact of Variations in Measurement Period Reporting for Electronic Clinical Quality Measures

**DOI:** 10.5334/egems.235

**Published:** 2018-07-19

**Authors:** Nicholas V. Colin, Raja A. Cholan, Bhavaya Sachdeva, Benjamin E. Nealy, Michael L. Parchman, David A. Dorr

**Affiliations:** 1 Oregon Health and Science University, US; 2 Kaiser Permanente Washington Health Research Institute, US

**Keywords:** Quality Improvement, Electronic Health Records, Data Collection, Health Services Research, Health Care Reform

## Abstract

**Objective:**

To understand the impact of varying measurement period on the calculation of electronic Clinical Quality Measures (eCQMs).

**Background:**

eCQMs have increased in importance in value-based programs, but accurate and timely measurement has been slow. This has required flexibility in key measure characteristics, including measurement period, the timeframe the measurement covers. The effects of variable measurement periods on accuracy and variability are not clear.

**Methods:**

209 practices were asked to extract and submit four eCQMs from their Electronic Health Records on a quarterly basis using a 12-month measurement period. Quarterly submissions were collected via REDCap. The measurement periods of the survey data were categorized into non-standard (3, 6, 9 months and other) and standard periods (12 months). For comparison, patient-level data from three clinics were collected and calculated in an eCQM registry to measure the impact of varying measurement periods. We assessed the central tendency, shape of the distributions, and variability across the four measures. Analysis of variance (ANOVA) was conducted to analyze the differences among standard and non-standard measurement period means, and variation among these groups.

**Results:**

Of 209 practices, 191 (91 percent) submitted data over three quarters. Of the 546 total submissions, 173 had non-standard measurement periods. Differences between measures with standard versus non-standard periods ranged from –3.3 percent to 14.2 percent between clinics (p < .05 for 3 of 4), using the patient-level data yielded deltas of –1.6 percent to 0.6 percent when comparing non-standard and standard periods.

**Conclusion:**

Variations in measurement periods were associated with variation in performance between clinics for 3 of the 4 eCQMs, but did not have significant differences when calculated within clinics. Variations from standard measurement periods may reflect poor data quality and accuracy.

## Introduction

Clinical trials are intended to facilitate the discovery of new knowledge, and the findings are used to drive health care decisions. These findings need to be useful [1] and trustworthy [2]; therefore, investigators of clinical trials must select reliable measures of outcomes for evaluation. Coster [3] notes that measures must match the study’s objective, population, and available instruments. Similarly, Velentgas et al. [4] provide a checklist with guidance and key considerations for measures in different types of studies, including considerations around measurement frequency and recall periods. For measures involving clinical outcomes, temporal aspects should be considered carefully, as there may be implications based on expected biological mechanisms. Furthermore, some conditions are acute while others are chronic, so considering the measurement period when evaluating treatments or interventions is important.

In studies related to health care quality and resource utilization outcomes, Velentgas and colleagues concluded that outcome measures should reflect the latest evidence-based clinical guidelines, and should be able to be measured from available data sources. Often we use electronic Clinical Quality Measures (eCQMs) in these trials, as these may facilitate measurement by using evidence-based definitions and data elements of clinical processes, structures, and outcomes found in most Electronic Health Record (EHR) systems. In quality payment programs such as Comprehensive Primary Care Plus (CPC+) or the Merit-based Incentive Payment System (MIPS), eCQMs are used to provide performance-based incentive payments. Standardized versions of eCQMs have specific definitions and logic encoded into the measure that define denominator criteria (patients eligible for the measure) and the numerator criteria (patients who are receiving a particular intervention). An eCQM is implemented by mapping these data elements and logic from the specifications against the appropriate clinical data in the EHR system. The numerator and denominator are then used to compute a performance percentage.

As part of a national effort to examine health care quality, the Agency for Healthcare Research and Quality launched a set of clinical trials in 2015 known as the EvidenceNOW initiative. EvidenceNOW is comprised of a group of regional research collaboratives that focus on heart health-related Quality Improvement (QI) trials in small to medium sized primary care practices. The QI focus was on four standardized eCQMs related to cardiovascular disease (CVD) risk factors, aligned with the “ABCS” measures from the U.S. Department of Health and Human Services’ “Million Hearts” initiative: aspirin use in high risk patients (CMS164; NQF0068), blood pressure control (CMS165; NQF0018), cholesterol management (CMS347; NQF pending), and smoking cessation counseling (CMS138; NQF0028). By helping primary care clinics improve their QI capacity with a focus on CVD risk factors, EvidenceNOW aims to achieve at least 70 percent performance on the ABCS measures, and potentially reduce the number of heart attacks and strokes in the United States.

As a regional collaborative within EvidenceNOW, the Healthy Hearts Northwest (H2N) collaborative is comprised of primary care practices across three states: Washington, Oregon, and Idaho. Practices were asked to submit data for the ABCS measures from their EHRs or related Health Information Technology (HIT) tool every 3 months with a standard (12-month) measurement period for each submission. While the numerator and denominator specifications of each eCQM generally aligned with the standardized definitions from the Centers of Medicare and Medicaid Services (CMS) and the National Quality Forum (NQF), the measurement periods in EvidenceNOW varied from those of most federal quality reporting initiatives, which require a once yearly measurement with either a 90-day or 1-year measurement period. The ability of practices’ EHRs to calculate a standard (12-month) measurement period on a rolling-basis varied; many systems had trouble meeting requirements defined by federal programs [5].

As with any secondary use of EHR data, there are many known challenges around data limitations and data quality, including those related to the completeness, correctness, and currency (timeliness) [6]. When EHR data from several organizations are used in clinical trials, the variation in clinical data available in information systems must be accounted for [7]. This variation has led to concerns about the validity of eCQM implementations, and highlighted difficulties when comparing performance among sites. In previous work, we found that implementations of the same eCQM using distinct value set specifications, and older versus newer versions of measure definitions, led to variations in the calculated prevalence of patients at-risk for key conditions, and in some cases led to variations in eCQM performance [8,9].

There is currently a lack of methodological literature around understanding the variation due to inability to standardize measurement periods in eCQMs. In this study, our objective was to understand the impact of varying measurement period on the calculation of eCQMs in the H2N EvidenceNOW collaborative.

*Our hypothesis was that eCQM submissions for standard measurement periods (12 months) versus non-standard measurement periods (e.g., 3, 6, or 9 months) would lead to differences in eCQM performance*.

## Methods

### Overview

As a component of participation in H2N, 209 participating practices were asked to extract data for the ABCS measures from their EHRs on a quarterly basis and report their aggregated values for the numerators and denominators using a standard (12-month) measurement period for each measure. Practices reported their quarterly eCQM data using an online REDCap survey, a secure web-application for online surveys and databases. Although we requested a 12-month measurement period for eCQM submissions, practices were asked to specify the measurement period used for quarterly submissions by choosing one of the following options: 1 year, 9 months, 6 months, 3 months and other. Table 1 provides the definitions of the ABCS measures.

**Table 1:** ABCS Measure Definitions.

Measure	Definition

**“Appropriate Aspirin Use”** – CMS164v5; NQF0064	Percentage of patients 18 years of age and older who were diagnosed with acute myocardial infarction (AMI), coronary artery bypass graft (CABG) or percutaneous coronary interventions (PCI) in the 12 months prior to the measurement period, or who had an active diagnosis of ischemic vascular disease (IVD) during the measurement period, and who had documentation of use of aspirin or another antiplatelet during the measurement period.
Ischemic Vascular Disease (IVD): Use of Aspirin or Another Antithrombotic	
**“Blood Pressure Control”** – CMS165v5; NQF0018	Percentage of patients 18–85 years of age who had a diagnosis of hypertension and whose blood pressure was adequately controlled (<140/90 mmHg) during the measurement period.
Controlling High Blood Pressure	
**“Cholesterol Management by Statin Therapy”** – CMS347v0; Quality ID 438	Percentage of the following patients-all considered at high risk of cardiovascular events-who were prescribed or were on statin therapy during the measurement period: Adults aged >= 21 years who were previously diagnosed with or currently have an active diagnosis of clinical atherosclerotic cardiovascular disease (ASCVD); OR Adults aged >= 21 years who have ever had a fasting or direct low-density lipoprotein cholesterol (LDL-C) level >= 190 mg/dL or were previously diagnosed with or currently have an active diagnosis of familial or pure hypercholesterolemia; OR Adults aged 40–75 years with a diagnosis of diabetes with a fasting or direct LDL-C level of 70–189 mg/dL.
Statin Therapy for the Prevention and Treatment of Cardiovascular Disease	
**“Smoking Cessation” – CMS138v5; NQF0028**	Percentage of patients aged 18 years and older who were screened for tobacco use one or more times within 24 months AND who received cessation counseling intervention if identified as a tobacco user.
Preventive Care and Screening: Tobacco Use: Screening and Cessation Intervention	

### Data Collection

Practices submitted data for three quarters of the H2N trial (Quarter 4 2015: 1/1/2015–12/31/2015, Quarter 1 2016: 4/1/2015–3/31/2016, and Quarter 2 2016: 7/1/2015–6/30/2016). The aggregate data was reviewed and the measurement periods of the submissions were cleaned and assigned 1 of 5 categories: 3 months (85–95 days), 6 months (170–190 days), 9 months (260–280 days), 12 months (350–370 days), or other (any measurement outside of aforementioned range). The measurement periods were then grouped into non-standard (3, 6, 9 months and other) and standard (12 months) for further analysis. Submissions with a null value for measurement period were removed from analysis.

For comparison, we measured the impact of implementing the ABCS measures with variation in measurement periods (3, 6, 9, and 12 months) on the same set of patient-level clinical data using an eCQM calculation registry known as the Integrated Care Coordination Information System (ICCIS). The ABCS measures in ICCIS were implemented against EHR data extracted from three primary care clinics (two family medicine and one internal medicine). All three of these clinics are urban teaching sites, with a total of 226 active providers. Provider types include: fellow (9), physician (98), osteopath (2), nurse practitioner (8), resident (97), physician assistant (10), and registered nurse (2). The total number of patients seen between Quarter 4, 2015 and Quarter 2, 2016 was 32,263. These three clinics had data quality assessments completed on their clinical data to verify accuracy and completeness.

### Evaluation

We assessed the frequency of submissions and univariate analysis for the ABCS measures. Using the groupings of the measurement periods (standard and non-standard) for the three quarterly ABCS submissions (H2N clinics) and the standardized comparison practices (ICCIS clinical data), we compared the aggregate mean values of performance for the ABCS measures along with their delta values. Analysis of variance (ANOVA) was conducted to analyze the differences among standard measurement period and non-standard measurement period means, and variation between these groups.

## Results

**Figure 1:**
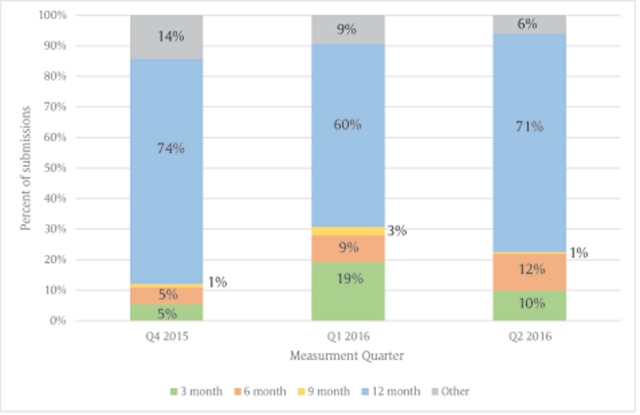
Frequency of Measurement Period Submitted by Quarter.

Of the 209 practices, 191 (91 percent) submitted at least one measure over the three quarters, yielding 546 total submissions (i.e. measure-quarters). 68 percent of the quarterly data submissions were reported with a standard measurement period and the rest varied. Of the 546 total submissions, only 463 included data for the Aspirin measure, 528 for Blood pressure, 137 for Cholesterol, and 465 for Smoking. Only 119 submissions included data for all four metrics, and of those, 102 (86 percent) were submitted with a standard measurement period. Figure 1 shows the frequency of measurement period submitted by quarter, between Quarter 4, 2015 to Quarter 2, 2016.

Of the 191 practices who submitted data, 88 (46 percent) were in a rural setting, while 103 (54 percent) were considered urban. 107 (56 percent) practices were part of a health system, while 84 (44 percent) were identified as independent. The average number of clinicians in each practice were 5.9 (range 1–43) and average number of patient visits per week were 251 (range 10–1100).

Quarter 4 of 2015 showed the greatest number of submissions with a standard measurement period with 134 submissions (74 percent). Quarter 1, 2016 showed a slight increase in eCQM submissions with a 3-month measurement period from the previous quarter (10 in Q4 2015 to 35 in Q1 2016).

### Submissions per Vendor

Table 2 lists the EHR vendors represented in H2N, and the frequency that were able to report standard and non-standard measurement periods. Of the 191 practices that submitted at least one measure over the three quarters of collected data (regardless of measurement period), only 165 (86 percent) of practices were able to submit any of their eCQM data with a standard measurement period. 13 practices had an EHR vendor that was not able to submit data with a standard measurement period, and of those, 6 (46 percent) of the practices were on Greenway Medical (Prime Suite, SuccessEHS). Practices using some vendors (e.g., Allscripts, Athenahealth) always had a submission with a non-standard measurement period, whereas other vendors (e.g., Amazing Charts) had up to 86 percent practices that were able to report standard measurement periods for all submissions.

**Table 2:** Counts of Practices Reporting Standard vs. Non-standard Measurement Periods by EHR Vendor Utilization.

Vendor	Total count of practices	% of practices with any non-standard measurement period submission	% of practices with at least 2 standard measurement period submissions	% of practices with all 3 standard measurement period submissions

Allscripts	2	100%	0%	0%
Amazing Charts	7	14%	86%	86%
Athenahealth	5	100%	60%	0%
Care360	1	100%	0%	0%
Cerner	5	80%	40%	20%
eClinicalWorks	12	75%	75%	25%
e-MDs	7	57%	86%	43%
EPIC	60	45%	77%	55%
GE/Centricity	17	94%	71%	6%
Greenway Medical	19	63%	95%	37%
McKesson/Practice Partner	5	80%	100%	20%
NextGen	9	67%	67%	33%
Practice Fusion	7	86%	29%	14%
Sage/Vitera	3	100%	0%	0%
SOAPware	3	33%	100%	67%
Other	3	100%	67%	0%
Total	165	63%	73%	37%

For each measure, Table 3 displays the denominator mean and differences between performance means in H2N clinic submissions with standard versus non-standard measurement periods. For comparison, mean performance data for the ABCS measures implemented in ICCIS are also displayed. Of the H2N submissions, the performance mean for the standard measurement periods were greater than that of the non-standard measurement periods for the aspirin and cholesterol management measures. The cholesterol measure showed the greatest difference for the performance mean between the standard and non-standard measurement periods with a delta value of 14.2 percent. Compared to the set of clinical data from ICCIS, the impact of variations in measurement period from standard to non-standard shows that non-standard measurement periods had greater performance means for three out of four measure, with delta values ranging from –1.55 percent to.58 percent.

**Table 3:** Reported Denominator and Performance for ABCS Measures within H2N and Standardized Comparison Submission.

eCQM Measure	H2N submissions (N = 191 practices)				Comparison submissions (N = 3 practices)			

	Denominator Mean (range)^1^	Performance Mean (Standard)	Performance Mean (Non-standard)	Delta^2^	Denominator Mean (range)	Performance Mean (Standard)	Performance Mean (Non-standard)	Delta

Use of aspirin (CMS164)	262.13 (1–2939)	70.3% (N = 320)	64.8% (N = 143)	5.5%	778.675 (229–1154)	76.09% (N = 10)	75.51% (N = 30)	.58%
Controlling high blood pressure (CMS 165)	962.44 (5–7810)	61.3% (N = 364)	64.6% (N = 164)	–3.3%	1983.4 (698–2770)	62.15% (N = 10)	63.70% (N = 30)	–1.55%
Cholesterol Management by Statin Therapy (CMS 347)	562.50 (2–3070)	68.9% (N = 111)	54.7% (N = 126)	14.2%	1301.85 (487–1797)	68.64% (N = 10)	69.37% (N = 30)	–.73%
Tobacco cessation intervention (CMS138)	2041.61 (6–22699)	71.2% (N = 316)	71.7% (N = 149)	–.5%	7071.5 (2854–10599)	70.91% (N = 7)	71.62% (N=21)	–.71%

^1^ Denominator Mean (range): The average number of patients reported per measure across both standard and non-standard measurement periods. ^2^ Delta: The difference in performance mean between the standard and non-standard measurement periods.

**Figure 2:**
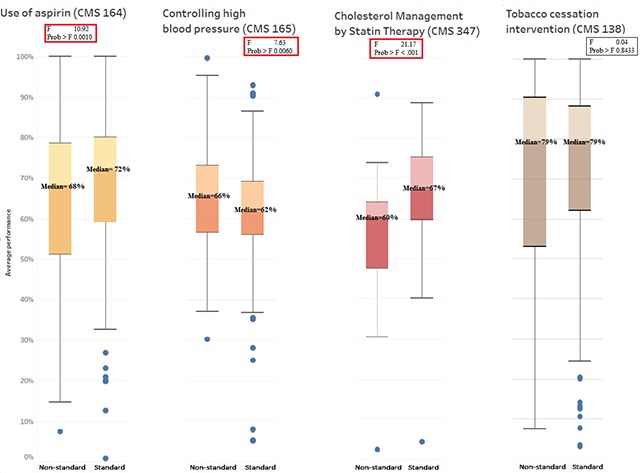
Non-standard vs. Standard Measurement Period Box-and-whisker Plots of eCQM Performance in H2N Submissions.

In Figure 2, the box-and-whisker plots for the four measures show greater variation in the non-standard measurement periods. ANOVA testing shows that the differences in variation for three of the four measures (aspirin, blood pressure, and cholesterol) were statistically different between standard and non-standard measurement periods.

## Discussion

We identified that the variations between standard and non-standard measurement periods resulted in statistically significant differences in the performance means of the ABCS eCQMs. The median performance values and range of values also differed the greatest for the non-standard measurement period submissions. The submissions with non-standard measurement periods had larger variation in their performance values for three out of four measures (aspirin, blood pressure and cholesterol) than those of the standard measurement period.

In assessing the number of submissions per quarter we highlighted gaps in reporting of the measures using the standard measurement period. This might reflect gaps in the implementation of the ABCS eCQMs in many practices’ EHRs. EHR vendors have typically implemented eCQMs as measures of accountability and not as measures for quality improvement. Asking practices to submit data for these measures with the requested standard measurement period on a rolling quarterly basis often is not an option of the reporting function from the EHR. Many EHR vendors build their measurement period with limited flexibility to change their date ranges and are often built with only year-to-date measurement periods. This is likely why we see an increase in the number of submissions for the 3-month measurement period for Q1 of 2016 and slight increase in 6-month measurement period for Q2 of 2016. Furthermore, we see that EHR vendors’ capabilities vary amongst themselves; for instance, 95 percent of practices using Greenway were able to report at least two submissions with standard measurement periods, while only 37 percent were able to report all three submissions with a standard measurement period. Meanwhile, of the practices using EPIC as their vendor, 55 percent were able to submit all three submissions with a standard measurement period while 45 percent of submissions used a non-standard measurement period. These variations show that EHRs likely have different functionalities even amongst cohorts of practices using the same systems.

In these practical implementations of eCQMs against EHR data, the types of significant variations we see can be attributed to continuous transformations in eCQM logic and definitions, and to vast differences in organizational structures of clinical data. This includes variation in measurement periods, and therefore, we may see differences in eCQM performance. In our analysis of the H2N submissions, we did find variation between standard and non-standard measurement periods, but it is hard to distinguish how much of the effect is due to measurement period when there are many other factors contributing to variation in eCQM implementations between clinics. To help understand the effect of the variation in measurement periods alone, as a comparison, the ABCS measures were implemented in ICCIS and measured against the same clinical data. In the H2N submissions, we did still see significant (p < 0.5 for 3 of 4) variation in performance with delta values from –3.3 percent to 14.2 percent between measures with standard versus non-standard measurement periods. However, using the same variations in measurement periods with the same clinical dataset from ICCIS yielded differences in performance with deltas of –1.55 percent to.58 percent with no significant differences. These differences seen in the ICCIS data likely indicate that the storage of clinical data and query calculation differ between the standard and non-standard measurement periods and are not a reflection of changes in clinical care by providers.

Accounting for these differences can be important to understand the variations in eCQM data produced by practices, particularly for research and quality incentive programs. The variations in eCQM performance seen in H2N can have substantial impact on incentive payments for practices. In CPC+, practices that fail to meet the 50th percentile performance threshold of 63.60 percent for the blood pressure measure (CMS 165) may lose out on 4 to 8.33 percent of their incentive payments [10]; in regards to H2N the non-standard vs. standard measurement period submissions split that threshold, indicating the submissions with the standard measurement period (61.3 percent) would have missed out on the payment. In the scope of H2N, the standard measurement period submissions for the aspirin measure (CMS 164) met the 70 percent goal of Million Hearts [11], while the submissions with a non-standard measurement period (64.8 percent) were below the Million Hearts goal. Future collections of EHR data should include metadata for how it is generated, including details about measurement periods. Standardized measures need to be implemented across EHRs to accurately assess health care quality across the health care system. One policy suggestion is to request EHR vendors to provide flexible reporting periods within their builds to enhance quality improvement activities. Another suggestion is to move towards a centralized HIT system or tool for eCQM calculation (e.g., PRIME, other registries) to avoid the vast variation created by local EHR customizations and eCQM implementations.

In using the practices’ native EHR functionality to produce the measures we see there are variations in the collected data. The direct influences of these variations are unknown. However, two potential factors should be identified. First, the reporting of eCQM data with a standard measurement period relied on practices accurately self-reporting their data. Practices could choose to report their data with other measurement periods. Second, practices’ selection of EHR vendors was not controlled in the study; this could impact the overall capabilities of practices to meet the demands of submitting data with a standard measurement period. Despite these variations that are seen in the collected data, the benefit is that we had higher rates of data submissions for the official, standardized measures developed by the Centers for Medicare and Medicaid (Aspirin, Blood Pressure, and Smoking) compared to EvidenceNOW’s novel measure (Cholesterol). Other EvidenceNOW cooperatives extracted data from participating practices’ EHRs and computed the measures in a central quality measure calculation registry. In H2N, we focused on the concept of teaching the practices how to sustain the methodologies they implemented throughout the project, and avoided this data extraction process. However, this did lead to variation in Aspirin and Cholesterol performance by measurement period, which is unexplained.

## Conclusion

Through the comparison of the standard and non-standard measurement periods, we identified that performance means of the H2N submissions for the ABCS measures varied significantly for three of the four measures (aspirin, blood pressure and cholesterol). Additionally, we identified that amongst EHR vendors, variations exist across the same vendors in their ability to calculate standard measurement periods. These differences in capabilities to run standard measurement periods are an indication of the various eCQM implementations that are present in the same EHR vendors. Therefore it is our recommendation that EHR vendors: (1) provide standard measurement periods and/or flexible measurement periods across eCQMs implementations; (2) include metadata for eCQM calculations, including details about specifics of the measurement period; and (3) collaborate with developers, payers, informaticians and policy makers to support a sustainable model to provide a centralized eCQM calculation.
